# Building a minimal and generalizable model of transcription factor–based biosensors: Showcasing flavonoids

**DOI:** 10.1002/bit.26726

**Published:** 2018-05-24

**Authors:** Heykel Trabelsi, Mathilde Koch, Jean‐Loup Faulon

**Affiliations:** ^1^ Micalis Institute, INRA, AgroParisTech University of Paris‐Saclay Jouy‐en‐Josas France; ^2^ Systems and Synthetic Biology Lab, CEA, CNRS, UMR 8030, Genomics Metabolics University Paris‐Saclay Évry France; ^3^ SYNBIOCHEM Center, School of Chemistry, Manchester Institute of Biotechnology University of Manchester Manchester UK

**Keywords:** biosensor, copy number, flavonoids, model, pinocembrin, transcription factor

## Abstract

Progress in synthetic biology tools has transformed the way we engineer living cells. Applications of circuit design have reached a new level, offering solutions for metabolic engineering challenges that include developing screening approaches for libraries of pathway variants. The use of transcription‐factor‐based biosensors for screening has shown promising results, but the quantitative relationship between the sensors and the sensed molecules still needs more rational understanding. Herein, we have successfully developed a novel biosensor to detect pinocembrin based on a transcriptional regulator. The FdeR transcription factor (TF), known to respond to naringenin, was combined with a fluorescent reporter protein. By varying the copy number of its plasmid and the concentration of the biosensor TF through a combinatorial library, different responses have been recorded and modeled. The fitted model provides a tool to understand the impact of these parameters on the biosensor behavior in terms of dose–response and time curves and offers guidelines to build constructs oriented to increased sensitivity and or ability of linear detection at higher titers. Our model, the first to explicitly take into account the impact of plasmid copy number on biosensor sensitivity using Hill‐based formalism, is able to explain uncharacterized systems without extensive knowledge of the properties of the TF. Moreover, it can be used to model the response of the biosensor to different compounds (here naringenin and pinocembrin) with minimal parameter refitting.

## 1 INTRODUCTION

Trends in metabolic engineering approaches to produce bio‐based chemicals in cell factories are still under continuous improvements. The main developments include overexpressing the enzymes of the rate‐limiting steps (Tai & Stephanopoulos, 2013), deletion of competing pathways (Stephanopoulos, 2012), balancing cofactor and precursor metabolites (Lan & Liao, 2012; Singh, Cher Soh, Hatzimanikatis, & Gill, 2011), implementing synthetic feedback loops (Dunlop, Keasling, & Mukhopadhyay, 2010; Harrison & Dunlop, 2012), and biosensor‐based dynamic regulation (Xu, Li, Zhang, Stephanopoulos, & Koffas, 2014).

One current challenging task is to set up a reliable method to screen for the best producing strains among a wide genetic diversity. The use of biosensors responsive to intracellular chemicals has opened doors to solving this pressing issue. Such sensory–regulatory devices, mainly transcription factors (TFs), have successfully been used to detect the presence of metabolites, but also for quantification and even high‐throughput screening (Pfleger, Pitera, Newman, Martin, & Keasling, 2007). Furthermore, biosensors can also play an important role in regulating pathway fluxes by sensing the level of a key intermediate and then promoting its synthesis or its downstream conversion (Xu et al., 2014). To overcome the limited number of naturally occurring metabolite‐responsive TFs available, progress has been made through their heterologous use, which includes transplantation of prokaryotic transcriptional activators into the eukaryotic chassis (Skjoedt et al., 2016). Additionally, it was recently shown that it is possible to expand the detection abilities by adding one or more enzymatic steps to transform a nondetectable compound into a detectable one (Delépine, Libis, Carbonell, & Faulon, 2016; Libis, Delépine, & Faulon, 2016). This latest tool considerably expands the scope of chemicals that can be sensed via transcriptional regulators.

**Figure 1:**
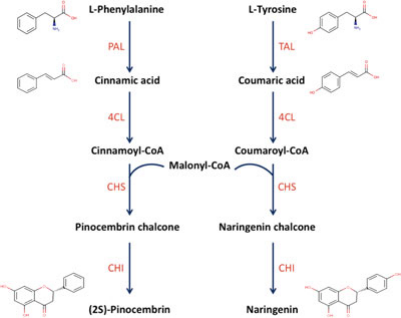
Pinocembrin biosynthesis pathway. PAL, TAL 4CL, CHS, and CHI refer to phenylalanine ammonia lyase, tyrosine ammonia lyase, coumarate‐CoA ligase, chalcone synthase, and chalcone isomerase, respectively [Color figure can be viewed at wileyonlinelibrary.com]

One of the interesting metabolic pathways implemented with relative success is the flavonoid pathway (Fehér et al., 2014). The industrial demand for some flavonoids is increasing, and among the top promising chemicals is (2*S*)‐pinocembrin, which is a plant secondary metabolite and the main starting point for the synthesis of other flavonoid molecules. This compound has a broad range of interesting characteristics such as antioxidant (Rasul et al., 2013), antibacterial (Weston, Mitchell, & Allen, 1999), antifungal (Peng et al., 2012), inhibitor of atherosclerosis (Yang et al., 2013), and neuroprotection in neurodegenerative diseases (Liu et al., 2012; Liu, Gao, Yang, & Du, 2008). To produce pinocembrin from glucose, four heterologous genes have to be implemented in *Escherichea coli* . First, phenylalanine ammonia lyase converts phenylalanine into cinnamic acid, which is then converted by coumarate‐CoA ligase into cinnamoyl‐CoA. Then, chalcone synthase condensates cinnamoyl‐CoA and three molecules of malonyl‐CoA to produce pinocembrin chalcone, which will be then converted into pinocembrin through chalcone isomerase (Figure 1). As of today, pinocembrin is produced at a low titer from glucose (Wu, Du, Zhou, & Chen, 2013; only 40 mg/L), and work still needs to be carried out to increase productivity, most likely through the building of combinatorial libraries with various enzyme sequences and regulatory elements (promoters, ribosome binding sites [RBSs]). Such libraries could be quickly screened with a pinocembrin biosensor, where the level of the reporter gene (i.e., fluorescence) is proportional to the pinocembrin titer.

Chemical structure similarity considerations of detectable flavonoids led us to choose as our candidate FdeR TF, a transcriptional activator‐based biosensor from *Herbaspirillum seropedicae* SmR1, shown to respond to naringenin (Marin et al., 2013; Siedler, Stahlhut, Malla, Maury, & Neves, 2014). Here, we have focused on developing and modeling the FdeR TF to shed light on the way we could design TF‐based biosensors to overcome issues of measurable quantification of metabolite production and to monitor an adequate sensing response. We have built different constructs varying most notably in plasmid copy number, changing both the concentration of the TF and the number of binding sites for the activated complex, and modeled the impact of this varying number on the sensitivity of the response. We provide a modeling strategy based on Hill functions to understand the impact of plasmid copy number and compound binding affinity to FdeR on our biosensor behavior, for both the dose–response and time curves, for a TF that has not been well characterized before.

## 2 MATERIALS AND METHODS

### 2.1 Plasmids and strains

All plasmids and strains used in this study are listed in the Supporting Information Table I. *E. coli* strain DH5 (Life Technologies, Darmstadt, Germany) and Mach 1 strain (Invitrogen Technologies, Carlsbad, CA) were used for cloning. *E. coli* strain BL21 (DE3) was used for enzyme expression. All the primers (P1–P9) were purchased from Eurofins genomics (Ebersberg, Germany) and are listed in the Supporting Information Table II. All our constructs were built by Gibson assembly using the NEBuilder HiFi DNA Polymerase Kit from New England Biolabs (Ipswich, Massachussets, MA). All plasmids were sequenced at the GATC Biotech (Konstanz, Germany). We performed all cloning and transformations as per standard protocols. Antibiotics were used at the following concentrations: ampicillin (Ap), 50 μg/ml; chloramphenicol (Cm), 25 μg/ml; kanamycin (Km), 30 μg/ml; and spectinomycin (Sp), 50 μg/ml.

### 2.2 Pinocembrin sensor library construction

Sixteen pinocembrin biosensors were constructed by varying the plasmid copy number and the RBS strength.

**Figure 2:**
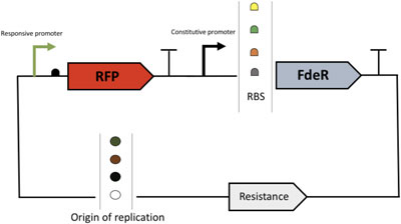
Schematic representation of the pinocembrin biosensor module. (a) Promotor, a ribosomal binding site precedes each gene. A terminator is located downstream of each gene. Resistance refers to chloramphenicol resistance, spectomycin resistance, ampicillin resistance, or kanamycin resistance. RFT, red fluorescent protein [Color figure can be viewed at wileyonlinelibrary.com]

First, primers P1 and P2 were used to amplify the plasmid backbones with different copy numbers from pACYCDuet‐1, pCDFDuet‐1, pETDuet‐1, and pRSFDuet‐1 (Supporting Information Table III). Second, the red fluorescent protein (RFP) under the control of the responsive promoter to pinocembrin was amplified from the plasmid pV20 (Supporting Information Table I) using the primers P3 and P4. Third, the FdeR TF with its constitutive promoter J23100 was amplified also from the plasmid pV20 with the four couples of primers P5/P9, P6/P9, P7/P9, and P8/P9 to generate the FdeR fragment with an RBS sequence 1, 2, 3, and 4, respectively. Finally, the 16 possible combinations were assembled in one step by Gibson assembly and confirmed by colonies PCR and sequencing (Figure 2). All the constructs of pinocembrin biosensors are highlighted in Supporting Information Table IV.

### 2.3 Biosensor dose–response characterization

For each biosensor strain, an isolated colony of BL21(DE3) harboring the appropriate plasmid was inoculated in 2 ml luria broth media (LB) containing the appropriate antibiotics and grown overnight at 37°C. The culture was then diluted 1:100 in fresh LB containing the appropriate antibiotics as well as different concentrations of pinocembrin, naringenin, or cinnamic acid (previously dissolved in ethanol) ranging from 1 to 500 µM. All the sensor cells were grown then for 24 hr with agitation at 37°C in microplate reader BioTek. Absorbance at 600 nm and fluorescence (Exc: 580 nm/Em: 610 nm) were measured. All experiments were repeated at least three times.

### 2.4 Chemical structure similarity

Compound InchI was obtained from Pubchem (Kim et al., 2016). Chemical structure analysis was performed using the KNIME (Berthold et al., 2009) analytics platform and RDKit nodes (Landrum, 2016). Tanimoto scores were computed using MACCS keys fingerprints (Durant, Leland, Henry, & Nourse, 2002).

### 2.5 Data normalization

RFP fluorescence reading was normalized by OD to obtain values that are proportional to per cell fluorescence. For fold change data, values obtained with inducers were divided by values obtained without inducers:

(1) $$\text{Fol}d_{ch\text{ange}}\left(\text{inducer}\right)=\;\frac{\text{RFP}/OD(\text{inducer})}{\text{RFP}/\text{OD}(\text{inducer}=0)}.$$

### 2.6 Simulation tools

All data analyses and simulations were run on R (version 3.2.3). Time evolution curves were simulated using the DeSolve package (version 1.14) and the rk4 algorithm, implementing the fourth‐order Runge–Kutta method. For random parameter sampling around the best fit, values were sampled from within ±1.96 standard deviation of the parameter estimate.

### 2.7 Parameter fitting

All parameters that could be found in the literature are highlighted in Table 1.

**Table 1:** Parameters, their values, and references

Parameter name	Parameter value	Parameter description	Method of obtention
*n_copy_* for 257	20	Copy number for the 257 construct	Novagen (Supplier)
*n_copy_* for 157	10	Copy number for the 157 construct	Novagen (Supplier)
*n_copy_* for 357	40	Copy number for the 357 construct	Novagen (Supplier)
*n_copy_* for 457	100	Copy number for the 457 construct	Novagen (Supplier)
*n*	1.84728055622324 ± 0.167856095615895 (dimensionless)	Cooperativity constant of the Hill model	Fitted on pinocembrin data
Ratio for 157	0.142356903084492 ± 0.0145559485207119 (AU)	Dynamic range of the construct divided by its copy number	Fitted on pinocembrin data
Ratio for 257	0.707131941851998 ± 0.0352622736990071 (AU)	Dynamic range of the construct divided by its copy number	Fitted on pinocembrin data
Ratio for 357	1.76685307850461 ± 0.0224367736516089 (AU)	Dynamic range of the construct divided by its copy number	Fitted on pinocembrin data
Ratio for 457	0.417451006235614 ± 0.0108399220317623 (AU)	Dynamic range of the construct divided by its copy number	Fitted on pinocembrin data
Correcting factor for naringenin	1.3	Correcting fold change factor	Estimated by averaging the correcting factors for individual constructs
*K_dsingle_*	887.835649014124 ± 120.681558027955 (µM)	The Hill constant, *K*_d_, for a single plasmid	Fitted on pinocembrin data
*K_m_* for pinocembrin	1 (dimensionless)	Ratio between the binding constants of the inducer and the transcription factor	By definition
*K_m_* for naringenin	2.14168405285072 ± 0.206020050654161 (dimensionless)	Ratio between the binding constants of the inducer and the transcription factor	Fitted on naringenin data
*n_tf*	2 (dimensionless)	The transcription factor forms dimers	Naringenin dose–response reference

The other parameters (*n*, *K*_m_, and $K_{d_{\text{single}}}$) were fitted using the nls (nonlinear square, from Package stats version 3.2.3) function using weighted least squares and the port algorithm (Dennis, Gay, & WalshWelsh, 1981), which allows for boundaries on the search space.

The time evolution parameters ($k_{\text{deg}}$ and α) were fitted using the optim function (from Package stats version 3.2.3, using the L‐BFGS‐B method implementing the Limited‐Memory Broyden Fletcher Goldfarb Shanno Algorithm, which is a quasi‐Newton method). Model function parameters were fitted locally to each data set of *n *= 3 replicates per data point unless otherwise stated. The final parameters used in the model are presented in Table 1 and Supporting Information Table V.

### 2.8 Sensitivity, fold change, and cooperativity of the different biosensors

To characterize the different biosensor dose–response curves, they were fitted to the following standard Hill function (Weiss, 1997):

(2) $$\text{Hill}(I)=\;\frac{{\left(I\right)}^{n}}{{{\left(K_{d}\right)}^{n}+\;(I)}^{n}}\times \text{ratio}+1,$$

where *I* is the concentration of the considered inducer (in µM); $K_{d}$ is the concentration that allows for half‐maximum induction (in µM as well), also termed IC_50_; *n* is the Hill coefficient that characterizes the cooperativity of the induction system; and ratio is the dynamic range (in arbitrary units).

## 3 RESULTS

### 3.1 Choice of the TF

Recently, Raman and colleagues were able to convert the intracellular presence of some flavonoids into a fitness advantage for the cell by combining the TtgR‐responsive domain (a regulatory gene of the multidrug efflux pump operon, *ttgABC*) to a TolC membrane protein (an *E. coli* outer membrane protein) necessary for survival under selective conditions. The strategy was successful in the screening of targeted genome‐wide mutagenesis for naringenin high‐producing strains (Raman, Rogers, Taylor, & Church, 2014). It is very useful in evolution experiments looking to enrich the culture with evolved variants and counter‐select the false positives but is not a first‐choice strategy when planning to screen libraries and pinpoint the response of every single clone. Our objective is to combine a TF with a fluorescent response to sense pinocembrin, which has not been previously reported. The use of Sensipath webserver (Delépine et al., 2016) has shown the need to transform pinocembrin to succinate or *S*‐adenosyl‐l‐homocysteine to be sensed by a transcription regulator. This would not be relevant from a metabolic engineering point of view, where the main objective is to increase the titer of the final product and not to consume it in some other auxiliary reactions even for a screening purpose. Direct detection in this case is more valuable. In a previous work, Siedler et al. (2014) have already characterized FdeR and Qdor, two TFs from *H. seropedicae* SmR1 and *Bacillus subtilis* shown to be responsive in *E*. *coli* to naringenin and kaempferol, respectively. Since these two compounds belong like pinocembrin to the flavonoid group, we have therefore performed a chemical structure similarity search in this family of chemicals. We have shown using the Tanimoto score that naringenin is the closest detectable compound to pinocembrin (see Section 2). We then decided to use the FdeR as a potential candidate to develop a pinocembrin biosensor (Table 2).

**Table 2:** Tanimoto scores for flavonoid compounds

Name	InchI	Tanimoto
Luteolin	1S/C15H10O6/c16‐8‐4‐11(19)15‐12(20)6‐13(21‐14(15)5‐8)7‐1‐2‐9(17)10(18)3‐7/h1‐6,16‐19H	0.8125
Apigenin	1S/C15H10O5/c16‐9‐3‐1‐8(2‐4‐9)13‐7‐12(19)15‐11(18)5‐10(17)6‐14(15)20‐13/h1‐7,16‐18H	0.8965
Genkwanin	1S/C16H12O5/c1–20‐11–6‐12(18)16–13(19)8–14(21–15(16)7–11)9–2‐4–10(17)5–3‐9/h2–8,17–18H,1H3	0.7812
Chrysin	1S/C15H10O4/c16–10‐6–11(17)15–12(18)8–13(19–14(15)7–10)9–4‐2–1‐3–5‐9/h1–8,16–17H	0.8965
Flavone	1S/C15H10O2/c16–13‐10–15(11–6‐2–1‐3–7‐11)17–14‐9–5‐4–8‐12(13)14/h1–10H	0.7241
Quercetin	1S/C15H10O7/c16–7‐4–10(19)12–11(5–7)22–15(14(21)13(12)20)6–1‐2–8(17)9(18)3–6/h1–5,16–19,21H	0.8125
Fisetin	1S/C15H10O6/c16–8‐2–3‐9–12(6–8)21–15(14(20)13(9)19)7–1‐4–10(17)11(18)5–7/h1–6,16–18,20H	0.7812
Kaempferol	1S/C15H10O6/c16–8‐3–1‐7(2–4‐8)15–14(20)13(19)12–10(18)5–9(17)6–11(12)21–15/h1–6,16–18,20H	0.8387
Galengin	1S/C15H10O5/c16–9‐6–10(17)12–11(7–9)20–15(14(19)13(12)18)8–4‐2–1‐3–5‐8/h1–7,16–17,19H	0.8387
Kaempferid	1S/C16H12O6/c1–21‐10–4‐2–8(3–5‐10)16–15(20)14(19)13–11(18)6–9(17)7–12(13)22–16/h2–7,17–18,20H,1H3	0.7647
Eriodictyol	1S/C15H12O6/c16–8‐4–11(19)15–12(20)6–13(21–14(15)5–8)7–1‐2–9(17)10(18)3–7/h1–5,13,16–19H,6H2/t13‐/m0/s1	0.9062
*Naringenin*	*1S/C15H12O5/c16–9‐3–1‐8(2–4‐9)13–7‐12(19)15–11(18)5–10(17)6–14(15)20–13/h1–6,13,16–18H,7H2*	*0.9655*
Isosakurametin	1S/C16H14O5/c1–20‐11–4‐2–9(3–5‐11)14–8‐13(19)16–12(18)6–10(17)7–15(16)21–14/h2–7,14,17–18H,8H2,1H3	0.875
Flavanone	1S/C15H12O2/c16–13‐10–15(11–6‐2–1‐3–7‐11)17–14‐9–5‐4–8‐12(13)14/h1–9,15H,10H2	0.7586
Pinocembrin	1S/C15H12O4/c16–10‐6–11(17)15–12(18)8–13(19–14(15)7–10)9–4‐2–1‐3–5‐9/h1–7,13,16–17H,8H2/t13/m0/s1	1

### 3.2 Biosensor design and construction

Marin et al. (2013) have identified the *fde* operon, associated with the degradation of aromatic compounds, mainly naringenin. The expression of this operon, under the regulation of the FdeR TF, is induced by naringenin. Thus, we have built a plasmid containing FdeR under a constitutive promoter and an RFP under the control of the responsive promoter from the *fde* operon. To build our combinatorial library to identify the best biosensors, we chose to build the constructs using four different plasmid copy numbers and four different RBS sequences for the FdeR gene (Figure 2).

### 3.3 Biosensor characterization

**Figure 3:**
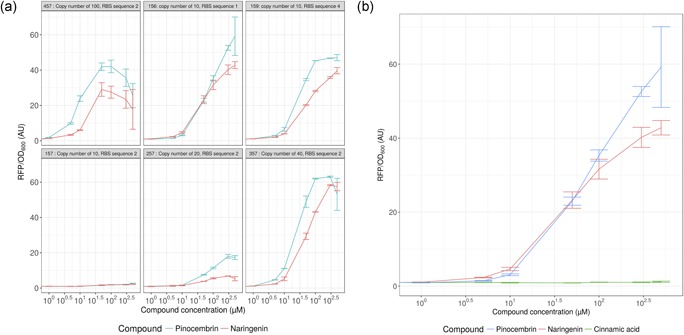
Dose responses of different biosensor constructs. (a) Constructs 457, 156, 159, 157, 257, and 357 were cultivated for 24 hr in the presence of increasing concentrations of pinocembrin (blue) and naringenin (red) ranging from 1 to 500 µM. Error bars are based on the standard deviation of a minimum of biological triplicate. (B) Biosensor 156 was cultivated for 24 hr in the presence of increasing concentrations of pinocembrin (blue), naringenin (red), and cinnamic acid (green) ranging from 1 to 500 µM. Error bars are based on the standard deviation of a minimum of biological triplicate [Color figure can be viewed at wileyonlinelibrary.com]

To benchmark our design, *E*. *coli* cells harboring the different constructs were grown for 24 hr in the absence and presence of increasing concentrations of pinocembrin or naringenin ranging from 1 to 500 µM, and red fluorescence was monitored in parallel with cell growth (Figure 3a). As expected, the different biosensor constructs were active in *E*. *coli* in the presence of naringenin. More interestingly, the different constructs were able to detect pinocembrin, and most of them have shown a high expression level of RFP exceeding in all cases the level of expression in the presence of naringenin. Moreover, FdeR appears to be more sensitive to pinocembrin than naringenin, as is evident from the steeper slope in Figure 3a. The results have shown that the minimal concentration of pinocembrin required to activate the TF ranges between 1 and 5 µM. The fold change is also shown to reach 60 folds in construct 156 for instance. In some cases, we highlighted a decrease in the fluorescence when we exceed 300 µM, which is probably due to the toxicity of the compound. This toxicity could also explain the difficulty in reaching high titer of pinocembrin in metabolic engineering experiments, where, as mentioned previously, the record is around 40 mg/L (Wu et al., 2013).

To validate this biosensor as a potential candidate for screening purposes, we tried to evaluate the specificity of FdeR. The sensor detects pinocembrin, but what about its biosynthesis intermediates? The work of Marin et al. (2013) has shown that this TF is not activated by phenylalanine or tyrosine, which are the precursors of pinocembrin and naringenin, respectively. Next, we investigated the effect of cinnamic acid, a key intermediate in the pinocembrin pathway. One of the most sensitive constructs (156, see list of constructs in the Supporting Information Table IV) was grown in the presence of increasing concentrations of cinnamic acid. The results show no detection of this compound (Figure 3b). As a conclusion, none of the major intermediates are detectable by FdeR. These data support our choice of using the FdeR biosensor as a tool to screen for pinocembrin‐ or naringenin‐producing cells.

### 3.4 Choosing an adapted modeling strategy

The FdeR TF has been studied in only a few previous publications (Marin et al., 2013; Siedler et al., 2014), which means although it is characterized enough to know which inducers will or might bind to it and induce a response in *E. coli*, there is no quantitative data available on the binding strengths of the inducers to the TF or of the complex to the promoter. We had the choice among three main modeling approaches: statistical physics model (Bintu et al., 2005; Rydenfelt, Cox, Garcia, & Phillips, 2014), mechanistic modeling (Berset, Merulla, Joublin, Hatzimanikatis, & Van Der Meer, 2017), or variations on Hill modeling (Qian, Huang, Jiménez, & Del Vecchio, 2017; Zucca, Pasotti, Mazzini, Cusella De Angelis, & Magni, 2012).

The statistical modeling approach makes use of extensive knowledge of the promoter, its inducer, and the TF. For instance, after reviewing several published works, Bintu et al. (2005) have highlighted the need of the following constants to model a transcriptional activator: different binding energies (RNA polymerase to the promoter, TF to the promoter, binding interaction between the two, RNA polymerase to the rest of the genome) as well as knowledge of the number of binding sites on the promoter or the number of promoters. This does not include yet the effect of the binding of the inducer to the TF or of the genetic context (e.g., if there is a DNA binding loop for repression). This kind of modeling has been applied to the Lac operon but remains elusive for less‐characterized systems such as our novel biosensor.

The mechanistic approach models all possible interactions in the system, or at least most, with an important number of parameters (Berset et al., 2017; 21 for the ArsR biosensor). This approach, although interesting, necessitates a lot of biological knowledge to minimize the number of unknown parameters, as well as knowledge of the interactions that do occur or not. This can therefore only be carried out in a relatively well‐known system. Those parameters are then fitted using system biology approaches and different optimization algorithms to avoid the main issue these models face: their sloppiness. Sloppiness characterizes the fact that different sets of parameters can model the data due to high interdependency between parameters. For example, when two parameters are used to model a forward and a backward reaction, which is actually at equilibrium given the time scale considered, an infinite number of parameters, whose ratio is the equilibrium constant of the reaction, will fit the data.

The Hill class of models does not necessitate a priori knowledge of the exact interactions between the species involved, although knowledge of the broad behavior of the interactions is necessary. This model has been, for example, extended to take into account resource competition (Qian et al., 2017), model both the binding with the inducer and complex binding to the promoter in the Lux system (Zucca et al., 2012) or any switch‐like behavior. Therefore, we decided to extend the Hill model to account for a key tunable parameter in synthetic biology: plasmid copy number. Our aim was to have a model with as little free parameters as possible that could account for this effect.

### 3.5 Effects of plasmid copy number that we intend to model

As can be seen in Figure 3a (or the Supporting Information Figure 1), increasing the copy number leads to increased production, as expected, except for construct 457 (very high copy number), showing a decline in production after 100 µM concentration of pinocembrin or naringenin. The constructs behave similarly for both compounds, although the biosensor is slightly more effective for pinocembrin detection than for naringenin detection, which is somewhat unexpected given that naringenin is its natural reported activator. Another interesting aspect is the effect of copy number on IC_50_ (concentration at which the biosensor reaches half‐maximum induction: it corresponds to *K_d_* in the standard Hill function). We can see that effect both in the figure where the induction starts at smaller concentrations of the inducer and in biosensor characterization (Supporting Information Table VI), where IC_50_ diminishes with copy number of the construct. We therefore decided to take that effect into account in our modeling effort.

### 3.6 Derivation of the dose–response model: Accounting for copy number

We aim to show here a dose–response model that can account for the effect of copy number on both pinocembrin‐ and naringenin‐responding constructs. We need to take into account two effects of plasmid copy number.

(1) The number of binding sites for the TF–inducer complex increases proportionally to the plasmid copy number, meaning that intuitively, to reach half‐maximum saturation, there needs to be that many more TF–inducer complexes.
(2) The TF is produced constitutively from the biosensor plasmid, so TF number scales with plasmid copy number.

We consider that all following processes are at equilibrium since chemical binding is a fast process compared with transcription and translation, and we are considering dose–response curves for the time being.

#### 3.6.1 Formation of the TF–inducer complex

We consider that the TF forms $n_{\text{tf}}$ multimers to derive our equations. According to the literature, FdeR forms dimers (Siedler et al., 2014), which means $n_{\text{tf}}\text{​}=\text{​}2$ will be used when simulating the data. Since the exact binding configuration with the inducers (naringenin and pinocembrin) is not known, we will start by considering the following equilibrium (Equation [3]). Other neglected cooperativity effects will be accounted for in the Hill cooperativity constant (Equations 4–6):

(3) $$n_{\text{tf}}T_{f}+I\underset{K_{\text{dis}}}{\Leftrightarrow }{T_{f}}^{c}.$$

Ignoring the order of binding, which is not important for the final equilibrium but only for the kinetics, not considered here, given the time scales of the considered processes, we have Equation (4), where $T_{f}$ is the concentration of the TF, ${T_{f}}^{c}$, of the TF complexes and $K_{\text{dis}}$ is the dissociation constant of the complex:

(4) $${T_{f}}^{c}=\;\frac{{{I\times T}_{f}}^{n_{\text{tf}}}}{K_{\text{dis}}}.$$

Note that $K_{\text{dis}}$ depends on the inducer considered here, either pinocembrin or naringenin, and it is the dissociation constant of the considered reaction. We first consider a classical Hill binding equation for the induction due to the TF before improving on this equation. The classical equation is Equation (5), where *n* is the cooperativity constant and ratio represents the maximum induction or the dynamic range. *K_d_* is the TF–inducer complex concentration needed for half‐maximum induction:

(5) $$P_{\text{fold}}=\frac{{\left({T_{f}}^{c}\right)}^{n}}{{\left(K_{d}\right)}^{n}+{\left({T_{f}}^{c}\right)}^{n}}\times \text{ratio}\times n_{\text{copy}}.$$

However, we want to consider the fact that the plasmid copy number changes the number of binding sites for the TF (proportional to the number of plasmids in our construct, as there might be cooperativity and therefore more than one binding site per plasmid). We propose the following modification to Equation (5), which accounts for the fact that to reach half‐maximum saturation of a higher number of binding sites, the number of binding complexes also needs to be that much higher:

(6) $$P_{\text{fold}}=\frac{{\left({T_{f}}^{c}\right)}^{n}}{{{\left(K_{d}\times n_{\text{copy}}\right)}^{n}\;+\;({T_{f}}^{c})}^{n}}\times \text{ratio}\times n_{\text{copy}}.$$

When replacing Equation (4) into Equation (6), we obtain

(7) $$P_{\text{fold}}=\frac{I^{n}}{{\left(\frac{K_{d}\times K_{\text{dis}}\times n_{\text{copy}}}{{T_{f}}^{n_{\text{tf}}}}\right)}^{n}+I^{n}}\times ratio\times n_{\text{copy}}.$$

Let $K_{m}=K_{\text{dis}}(\text{compound})/K_{\text{dis}}(\text{pinocembrin})$ in Equation (8) be the ratio between the dissociation constant of the compound of interest divided by the one for pinocembrin, where the dissociation constant in itself is unknown. Therefore, $K_{m}=1$ for pinocembrin and $K_{m}=K_{\text{dis}}(\text{naringenin})/K_{\text{dis}}(\text{pinocemb}\text{rin})$ for naringenin. Introducing this parameter allows us to only consider the difference of binding strength between FdeR and naringenin or pinocembrin instead of the absolute binding values, which would add one sloppy parameter to our model. Moreover, since the TF is produced under a constitutive promoter on the plasmid, we can assume it is produced proportionally to the plasmid copy number. The proportionality constant is included into the $K_{{\mathrm{dsingle}}_{\;}}$ constant, as well as $K_{\text{dis}}(\text{pinocembrin})$, leading to Equation (8). We can note here that given our hypothesis (number of TFs and binding sites scaling with the copy number), no effect would be obtained in our model if FdeR were not a dimer:

(8) $$P_{f\text{old}}=\frac{I^{n}}{{\left(K_{{\mathrm{dsingle}}_{\times }}K_{m}\times {n_{\text{copy}}}^{1-n_{\text{tf}}}\right)}^{n}+I^{n}}\times \text{ratio}\times n_{\text{copy}}.$$

### 3.7 Analysis of the dose–response model

#### 3.7.1 Model fitting of pinocembrin

The model was fitted to the data according to the procedure presented in materials and methods. The fitted parameters were $K_{{\mathrm{dsingle}}_{\;}}\mathrm{, n}$ and ratio, as by definition $K_{m}=1$ for pinocembrin. The obtained parameters are listed in Table 1. Since we intend to model the effect of copy number variations, we chose to use constructions sharing the same RBS sequence for our parameter fitting: 157, 257, 357, and 457.

**Figure 4:**
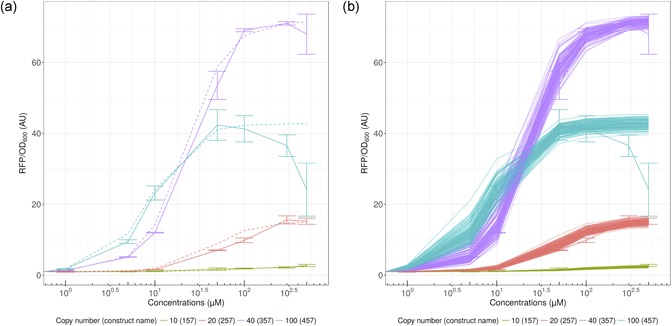
Model fitting to pinocembrin data for varying copy numbers. (a) Best fit parameters for pinocembrin. (b) 100 random simulations from parameter fitting for pinocembrin. Error bars represent standard deviation [Color figure can be viewed at wileyonlinelibrary.com]

We chose to represent both the best fit (Figure 4a) and 100 simulations (Figure 4b), where parameters were randomly sampled from the estimated distribution of parameters (see Section 2 for more details). We can see when looking at the random parameters that there is some leeway in the estimation, allowing for a rather wide dose–response curve. However, the expected behavior is maintained, even accounting for uncertainty in the estimation of the parameters. We chose to use the same cooperativity constant n, as well as the same $K_{{\mathrm{dsingle}}_{\;}}\text{,}$ constant, which would be the IC_50_ for a single plasmid, and hence its name. However, as mentioned in the data analysis section, the dynamic range does not scale proportionally with the plasmid copy number. For this reason, ratios varying from 0.14 to 1.76 were obtained and used in this study, instead of using a single parameter for this effect. This is due to a host of factors: higher plasmid copy number diverts more resources from the cell, the replication machinery is not the same for the different plasmids, which have different replication origins, and the cells do not divert resources to plasmids proportionally to their copies. Moreover, an interesting feature of the data is that production from the very high copy number construct (457) is initially higher than with the high copy number (357) until concentrations cross a threshold. We can imagine that the demand on the cell from our constructs becomes too high in the 457 construct, and the cell activates a “stress response.” This is observed when using both compounds for induction.

#### 3.7.2 Model fitting of naringenin

**Figure 5:**
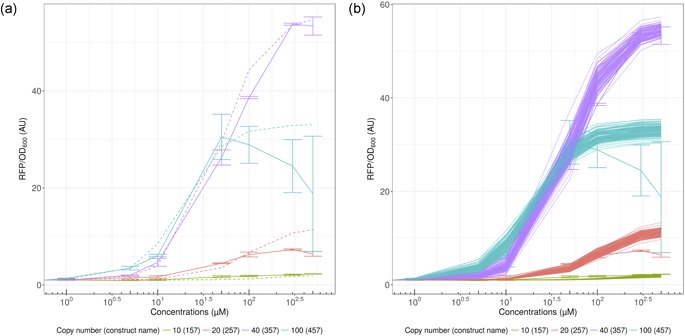
Model fitting to naringenin data for varying copy numbers. (a) Best fit parameters for naringenin. (b) Hundred random simulations from parameters fitting for naringenin. Error bars represent standard deviation [Color figure can be viewed at wileyonlinelibrary.com]

We were interested to determine whether the model could reproduce the features observed in the naringenin data: globally lower fold change of induction than for the pinocembrin induction, but the same overall behavior on sensitivity. Our aim was to account for the compound change using only our $K_{m}$ parameter, which represents the ratio between the dissociation constants of inducers to the TF ($K_{m}=K_{\text{dis}}(\text{naringenin})/K_{\text{dis}}(\text{pinocembrin})$). The results of this modeling strategy, when fitting only *K_m_*, give the results presented in Supporting Information Figure 2. However, as mentioned in Section 3.7.1, we chose to model fold change variation with a single parameter (multiplied by the copy number). Therefore, our model can capture changes in sensitivity due to both copy number increases and compound changes, but since this is included in our Hill function, variations of fold change at saturating amounts of substrates cannot be captured. Therefore, we added a correcting factor for all naringenin models, reducing all ratio values in our models by 1.3. This factor was chosen as a weighted average of correcting factors for the different constructs. The results obtained by this strategy can be seen in Figure 5a,b.

The global behavior of the biosensor is respected for all sensors, meaning that the same model does apply to this data. The shift in dose response can be explained by the *K_m_* parameter, which shifts the curve toward less sensitivity by doubling IC_50_. This is confirmed by the data for 357 and 457 constructs, which are the constructs with the least variability on IC_50_ estimation. We can also observe that the dynamic range is slightly lower, meaning that our modification of the ratio parameter by the same correcting factor is justified (for naringenin concentrations up to 100 µM). This could be explained by an effect that is not taken into account in our model, such as higher load, or some different toxicity between pinocembrin and naringenin. $K_{m(\text{naringenin})}$ is bigger than one, which means that the dissociation of FdeR dimer with naringenin is higher than the one with pinocembrin. In other words, at the same TF and inducer concentration, there is more TF bound with pinocembrin than would be with naringenin. This is surprising given the fact that FdeR was identified in the *fde* operon from *Herbaspirillum seropedicae*, which is involved and was identified for its implication in naringenin degradation. This means that we expected it to be evolved for naringenin detection, but that it detects pinocembrin at least as well.

All this indicates that our model, although very simple and based on broad knowledge of the sensor rather than precise chemical constant values, manages to successfully capture our system’s behaviour.

### 3.8 Time‐course model assumptions and derivation

Once we had a satisfying dose–response model, we chose to model the time‐dependent response of our biosensor, to determine the delay between the signal and the fluorescence production. We considered a relatively simple time‐course model, consisting of a production term and a degradation term for the protein. Results are presented in Appendix 1. This time‐course modeling partially allowed us to understand the impact of initial dilution on the biosensor’s behavior and emphasized the need to wait for it to reach steady state for it to be fully functional and decipher between different inducer concentrations. The shortcomings of this time‐course modeling confirm that although it is interesting to see the delay in response of the biosensor signal, modeling the dose–response curve is more important to show characteristics of the biosensor, such as changes to the dose–response curve when used for screening pinocembrin‐producing strains.

### 3.9 Leveraging our model for biosensor design improvement

**Figure 6:**
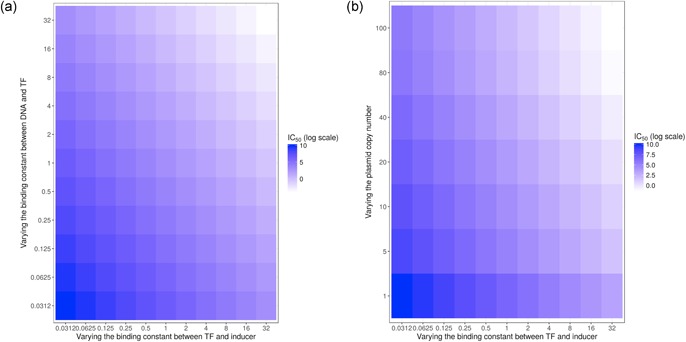
Effect on biosensor sensitivity of varying copy numbers, DNA, and transcription factor (TF) binding affinities or transcription factor and inducer binding affinities. Half‐maximum induction (IC_50_), used as a proxy for sensitivity, is represented in colors ranging from white (low IC_50_, high sensitivity) to dark blue (high IC_50_, low sensitivity) on a log scale. Binding constants are represented as fold‐change compared with current fitted constants. (a) Comparison of the effect of changing TF and DNA binding constants and TF and inducer binding constant. (b) Comparison of the effect of changing plasmid copy number and TF and inducer binding constant [Color figure can be viewed at wileyonlinelibrary.com]

Having constructed a satisfying dose–response model, it becomes interesting to use it to make predictions for future improvements of our design. We therefore considered three parameters that synthetic biologists can tune and study their effect on half‐maximum induction (IC_50_), used as a proxy for sensitivity. A higher IC_50_ means shifting the sensitivity of the biosensor toward higher concentrations and therefore can be used to screen higher producing strains. A lower IC_50_ means shifting it toward lower concentrations and more sensitivity to trace amounts of pinocembrin. The three parameters whose effects we decided to study are the following: plasmid copy number, DNA and TF binding strength, and TF and inducer binding strength. Plasmid copy number can easily be tuned by choosing the replication origin of the plasmid, DNA–TF affinity can be modified either by random mutagenesis of the promoter or by protein engineering (and measured through gel retardation assays), and TF–inducer affinity can be tuned by protein engineering. In Figure 6, we represent fold change compared with current fitted constants for TF and inducer binding strength. DNA and TF dissociation constant being captured by our Hill equation, it is proportional to our $K_{{\mathrm{dsingle}}_{\;}}$, constant, so the binding strength is proportional to the inverse of $K_{{\mathrm{dsingle}}_{\;}}$, and we are also representing fold changes around this constant. The copy number, on the other end, is represented as the desired value for copy number, as that can be achieved by choosing a correct replication origin to achieve the desired copy number. We can see in Figure 6a that increasing the binding constant between TF and DNA or TF and the inducer has similar consequences: increasing it leads to lower IC_50_ or higher sensitivity, whereas decreasing it leads to higher IC_50_, allowing one to detect higher titers of pinocembrin. This suggests that random mutagenesis at the promoter might be a better first approach to tune the biosensor’s behavior to an experimentalist’s needs, since it is easier to engineer rather than engineering the binding strength of the TF and its inducer, and both have similar consequences. Figure 6b, on the contrary, shows the impact of changing plasmid copy number or binding affinity of the TF for the inducer. As seen in our experimental data, increasing the copy number (which leads to higher expression) also increases sensitivity, allowing for better detection of the inducer but at lower concentrations. Reducing the copy number enables detection at higher titers, but reduces the fold change of the biosensor. On the contrary, augmenting the affinity of the TF to the inducer boosts sensitivity but does not allow differentiating different responses at high concentrations of the inducer. Therefore, our model suggests possibilities to further engineer our system, whether to sense high titers of pinocembrin to increase the biosensor’s sensitivity.

## 4 DISCUSSION

The use of TF‐based biosensors is expanding in many fields, ranging from environmental, biomedical to industrial biotechnology applications and more specifically as a fast and reliable screening tool to address the problems of high‐throughput limits of the other approaches (Dietrich, McKee, & Keasling, 2010; Eggeling, Bott, & Marienhagen, 2015). Some successful attempts have been reported describing strategies leading to the fine‐tuned response dynamics and dynamic ranges by engineering tunable biosensors (Chen, Xia, Lee, & Qian, 2017; Rogers et al., 2015). TFs have a ligand‐binding domain most likely to be promiscuous. In this study, we showcased the potential of chemical structure similarity scoring to select TF starting candidates to develop or engineer biosensors for small molecules. We have constructed a biosensor to detect pinocembrin with a fold change of around 60. FdeR appears unexpectedly to be more sensitive to pinocembrin than to naringenin, its natural effector, and has the required specificity to discriminate against the intermediates in the pinocembrin biosynthetic pathway. Indeed, the first report of this TF in Marin et al. (2013) identifies FdeR as the TF responsible for the regulation of a naringenin degradation operon. However, our experiments prove that FdeR senses pinocembrin at least as well, suggesting that this operon could also be involved in pinocembrin degradation. Two possible degradations pathways were identified by Marin et al. (2013) based on *in silico* analysis of the enzymes found in the operon. One started by opening the C‐ring of naringenin, whereas the other opened the A‐ring. In both cases, since pinocembrin differs from naringenin by a group on the B‐ring, it could also be degraded by these pathways. A recent study performed by Zhang et al. (2017) was also successful in generating a new biosensor for specific lactam compounds using a chemoinformatics approach inspired by small‐molecule drug discovery. Methodologies based on the structural analysis of compounds could offer an alternative to some heavy strategies based on the design of new TFs for nonnatural ligands (Looger, Dwyer, Smith, & Hellinga, 2003; Mandell & Kortemme, 2009; Schallmey, Frunzke, Eggeling, & Marienhagen, 2014) or by random mutagenesis (Tang, Fazelinia, & Cirino, 2008; Tang et al., 2013; Tang & Cirino, 2011).

To extend our knowledge of the rules governing the sensitivity, specificity, and dose responses of biosensors, we have also built different sensor constructs varying the copy number and the RBS to scan different response patterns that could serve as a template for modeling and to help extract rational understanding of the biosensor behavior.

Although simple, the model developed in this paper allows us to explain the behavior of our biosensor to both naringenin and pinocembrin with a single parameter that accounts for the binding variability between these two compounds and the TF. It also accounts for variations of copy number on the sensitivity of the biosensor starting from a simple idea: if there are more binding sites, there is a need for proportionally more activators to reach half‐maximum saturation. This is a simple but useful addition to the synthetic biology modeler’s toolbox when working on poorly characterized systems where more robust modeling approaches, such as mechanistic or statistical modeling, are not possible to use. Our model allows us to not only describe trends but also quantitatively correct values.

An interesting effect we managed to capture is the effect of copy number on IC_50_. This effect was already observed in a previous work of Zucca et al. (2012) although the authors did not investigate the link between copy number and IC_50_. Although they have an IC_50_ that increases with copy number (although the relationship is not linear), the way they model their binding renders a numerical comparison impossible.

In the present paper, we have two different effects when increasing copy number: we increase the number of binding sites (increasing IC_50_) but we also increase the number of available TFs, allowing for more binding even with less inducer, thereby reducing the IC_50_. According to our model, if the TF concentration was not increasing, we would also see a reduced sensitivity, as found in Zucca et al., which confirms our biosensor design idea.

As we have seen, the time evolution model is not fully satisfying. A few strategies could help make it closer to the data, but they all present the disadvantage of adding new free parameters: adding a lag time for protein production as the introduced dilution does not seem to be enough and add some toxicity or load effect when copy number, TFs, and inducers are in too great numbers. These were not implemented as our aim was to present a model with a minimal set of parameters that explained the data well enough.

Another interesting feature of our model is to suggest further modifications of our design depending on the desired application: increasing its sensitivity, its dynamic range, or being able to sense higher titers of pinocembrin, by capturing the effects of changing copy number, DNA–TF binding affinity, or TF–inducer binding affinity.

As a conclusion, we have presented a simple model with a minimal number of parameters that allows us to capture the effects of both copy number and inducer variations on our biosensors’ behaviors and most notably on sensitivity, which are effects that have not been addressed as such and especially never with such a simple formalism. This model, based on a simple Hill equation, has the advantage of being very versatile and easy to use on previously uncharacterized systems.

The development of the pinocembrin biosensor, its modeling, and understanding its behavior open doors to generate more transcription‐factor‐based biosensors to meet the increasing demands of screening and dynamically regulating metabolic pathways in industrial strains.

## CONFLICTS OF INTEREST

The authors declare that there are no conflicts of interest.

## AUTHORS’ CONTRIBUTIONS

H. T., M. K., and J.‐L. F. designed the study. H. T. designed, built, and characterized the biosensors. M. K. performed the chemical structure analysis, the mathematical analysis, and the modeling. All authors participated in the interpretation of the results and in the preparation of the manuscript.

## Supporting information

Supporting information

Supporting information
