# Supplementary material for: Building a minimal and generalizable model of transcription factor–based biosensors: Showcasing flavonoids
Source: Biotechnol Bioeng. 2018 May 24;115(9):2292–304. doi: 10.1002/bit.26726 (PMC6548992; doi:10.1002/bit.26726)
Supplement: Supplementary file 1 — Supporting information [file BIT-115-2292-s001.docx]

**Building a minimal and generalizable model of transcription-factor based biosensors: showcasing flavonoids**

Heykel Trabelsi,^1^**^*^** Mathilde Koch,^1^**^*^** Jean-Loup Faulon^1,2,3^

Affiliations:

1) Micalis Institute, INRA, AgroParisTech, University Paris-Saclay, 78350 Jouy-en-Josas, FRANCE

2) UMR 8030 Genomics Metabolics, Systems and Synthetic Biology Lab, CEA, CNRS, University of Evry-val-d’Essonne, University Paris-Saclay, Évry, FRANCE

3) SYNBIOCHEM Center, Manchester Institute of Biotechnology, School of Chemistry, University of Manchester, Manchester, UK

*** These authors contributed equally to this work**

Corresponding Author:

E-mail:[jean-loup.faulon@inra.fr](mailto:jean-loup.faulon@inra.fr)

**Appendix 1:**

**Time-course model assumptions and derivation**

Once we had a satisfying dose-response model, we chose to model the time-dependent response of our biosensor, to see the delay between the signal and the fluorescence production.

We consider a relatively simple time-course model, consisting of a production term and a degradation term for the protein. mRNA is not explicitly taken into account here as models explicitely taking it into account do not provide better fits to the data but add unnecessary parameters (results not shown).

$\frac{dRFP}{dt}= P\left( inducer, construct \right)-k\left( RFP \right)$ (9)

Equation 9, where *P (inducer, construct)* is a function that describes the production depending on the inducer concentration *I* and the chosen construct, and *k* is a term that encompasses both dilution and degradation processes. RFP here represents the RFP produced by an individual cell, therefore it corresponds to the normalized RFP data. Inducer concentration is considered to be the external inducer concentration.

Modeling dilution and degradation:

As can be seen in the time evolution of our constructs (Supplementary Fig. 3) represents time evolution of the construct 357 for OD (a) and RFP/OD (b)), dilution plays an important role during the first hours, which correspond to exponential growth phase. When cells reach stationary phase (after 30 to 40 thousand seconds, or 8 to 11 hours), RFP that has been accumulating also reaches a steady state concentration in the cell when degradation compensates for production. Therefore, we have to take into account both these phenomena when modeling the time response of our biosensors, instead of using a constant term for k as is usually done.

We therefore chose to model it that way:

$k\left( RFP \right)=\left( k_{dil}(t)+k_{\deg} \right)*RFP$ (10)

where $k_{\deg}$ is an unknown constant that will be fitted to the data and $k_{dil}(t)$ is a function that accounts for dilution, as we consider first-order degradation and not more complex mechanisms such as Michaelis-Menten degradation. Our aim here was not to fully account for this phenomenon, since we were focusing on characterising and modeling the effect of copy number. Therefore, we chose a logistic growth as a simple model for modeling bacterial growth:

$OD\left( t \right)= \frac{OD_{m}*OD_{0}*{exp}^{k\left( t \right)}}{OD_{m}+OD_{0}({exp}^{k\left( t \right)}-1)}$ (11.a)

$k_{dil}\left( t \right)= k*\frac{OD_{m}-OD_{0}}{OD_{m}+OD_{0}({exp}^{k\left( t \right)}-1)}$ (11.b)

This model was fitted to the OD on one set of experiments (construct 357, concentration of 100 µM of pinocembrin, (Supplementary Fig. 4A) and then used to model all other constructs (Supplementary Fig. 4B). The growth rates of different constructs are similar and independent of the resistance cassette chosen (Supplementary Figure 5). As we can see, this model represents rather well growth for low concentrations but is not built to account for toxicity at higher concentrations. Therefore, the first hours of the time evolution are imperfectly fitted as this was not fitted for individual constructs and experiments. The aim here was to use only one set of parameters for all constructs and experiments, where another approach would have been to fit the logistic growth model to all individual experiments and then use these parameters. Since the values we are interested in for a biosensor are steady state values, and more precisely does-response curves, using the same set of parameters for growth instead of separately fitting this model to OD data is not an issue. Moreover, these dilution parameters mostly account for what happens at the beginning of the induction, since it is obvious from the dilution equation that $\lim_{t\to} k_{dil}\left( t \right)= 0$. This confirms that using the same parameters is not an issue as the values we are interested in for a biosensor are steady state values.

Steady state- transfer function:

Before focusing on the production term that was our main interest in the previous sections, we will show how we derive dose-response equations from the time-course model.

As can be seen from the dilution equation, $\lim_{t\to} k_{dil}\left( t \right)= 0$. Therefore, at steady state,

$\lim_{t\to} RFP= \frac{P(inducer, construct)}{k_{deg}}$(12), which justifies why we could study steady-state without considering time evolution.

Production term:

The production term is classically derived as

$P= \alpha*basal*\left( 1+P_{fold}(inducer, construct \right))$(13)

Equation 13, where $\alpha$ takes into account the production, fluorescence intensity and gain of the measurement apparatus. $basal$ represents the basal production for the dose-response curve, or the production without induction: it is 1 when we are considering already normalized data (by fold change). The transfer function is the one that accounts for the variations between constructs and the effect of the inducer, which was developed and analyzed in the previous sections.

Therefore, the only 2 parameters that need fitting are $k_{deg}$and $\alpha$. This was done on construct 357, with a concentration of 100 µM of pinocembrin according to the procedure presented in materials and methods. We then simulated the expected dose-response from this time simulation and compared it to the steady-state model and the dose-response curve.

We can see in Supplementary Fig. 6(A, B) that the model and the data are in rather good agreement when steady-state behavior is reached, but that the initial dilution is not well taken into account. This is even worse when no time-dependent dilution is included (data not shown) as the curve has to be monotonous and therefore cannot account for the drop in normalized fluorescence. The dose response curves are also in agreement, although the time-evolution is still slightly above the data because of the exponential modeling of the degradation.

The same parameters were then used to simulate the time evolution of the same construct with an induction by naringenin instead of pinocembrin and the results are presented in Supplementary Fig. 7(A, B). The overshooting tendency of the simulated data is even more pronounced. This time-course modeling partially allows us to understand the impact of initial dilution on the biosensor’s behavior, and emphasizes the need to wait for it to reach steady-state in order for it to be fully functional and decipher between different inducer concentrations. This initial dilution, although not fully accounted for here, partly explains the delay between the signal (present in the media) and the biosensor’s full response. The model also allows us to suggest biological consequences of our constructs that were not accounted for and could be of interest to explain more thoroughly this time-delay: a lag-time in protein production or some toxicity effects of our constructs. The shortcomings of this time-course modeling confirm that although it is interesting to see the delay in response of the biosensor signal, modeling the dose-response curve is more important to show characteristics of the biosensor such as changes to the dose-response curve when used for screening pinocembrin-producing strains.

**
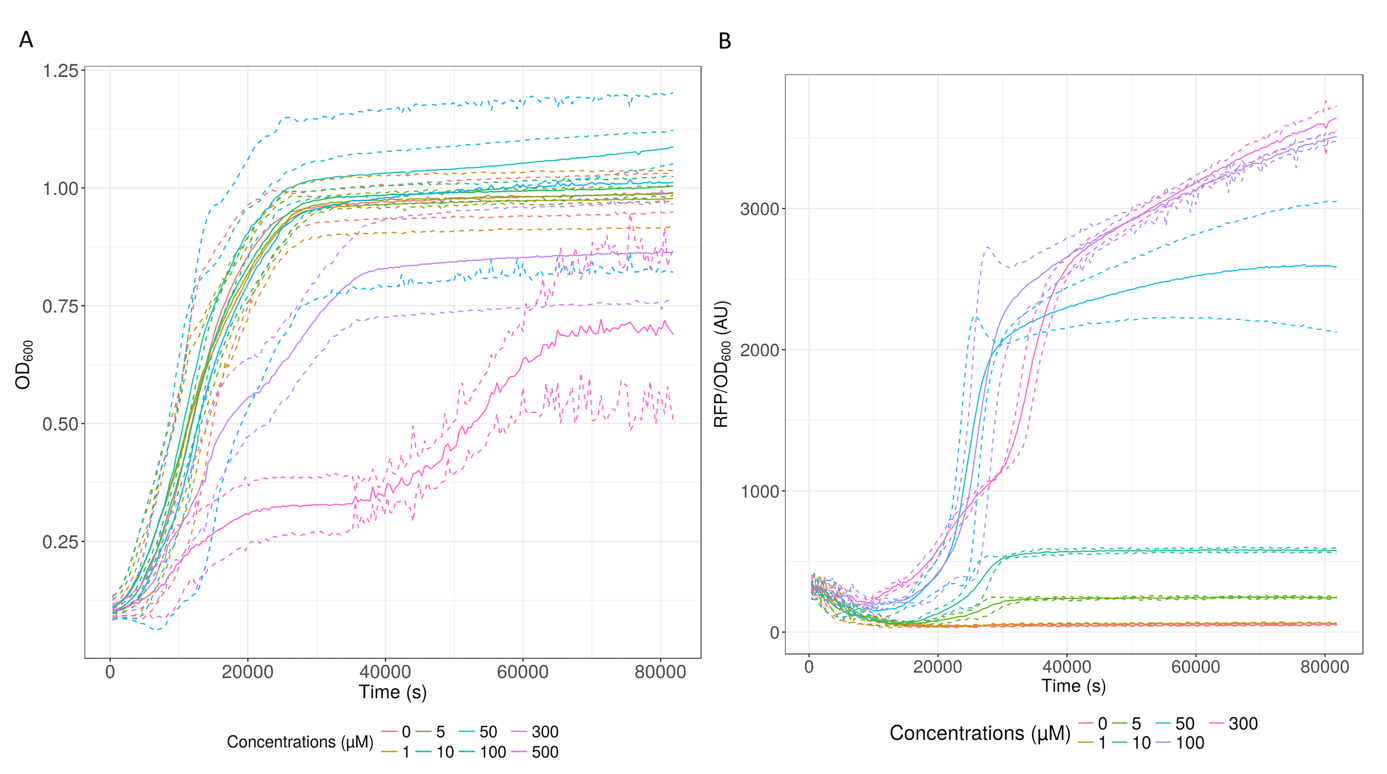
**

**Supplementary figure 3. Time course modeling of the construct 357. (A)** OD data. **(B)** RFP divided by OD data. Solid lines represent mean of the experimental data and dashed lines 95% confidence intervals. Different colors represent varying concentrations as indicated on the Figure.

**
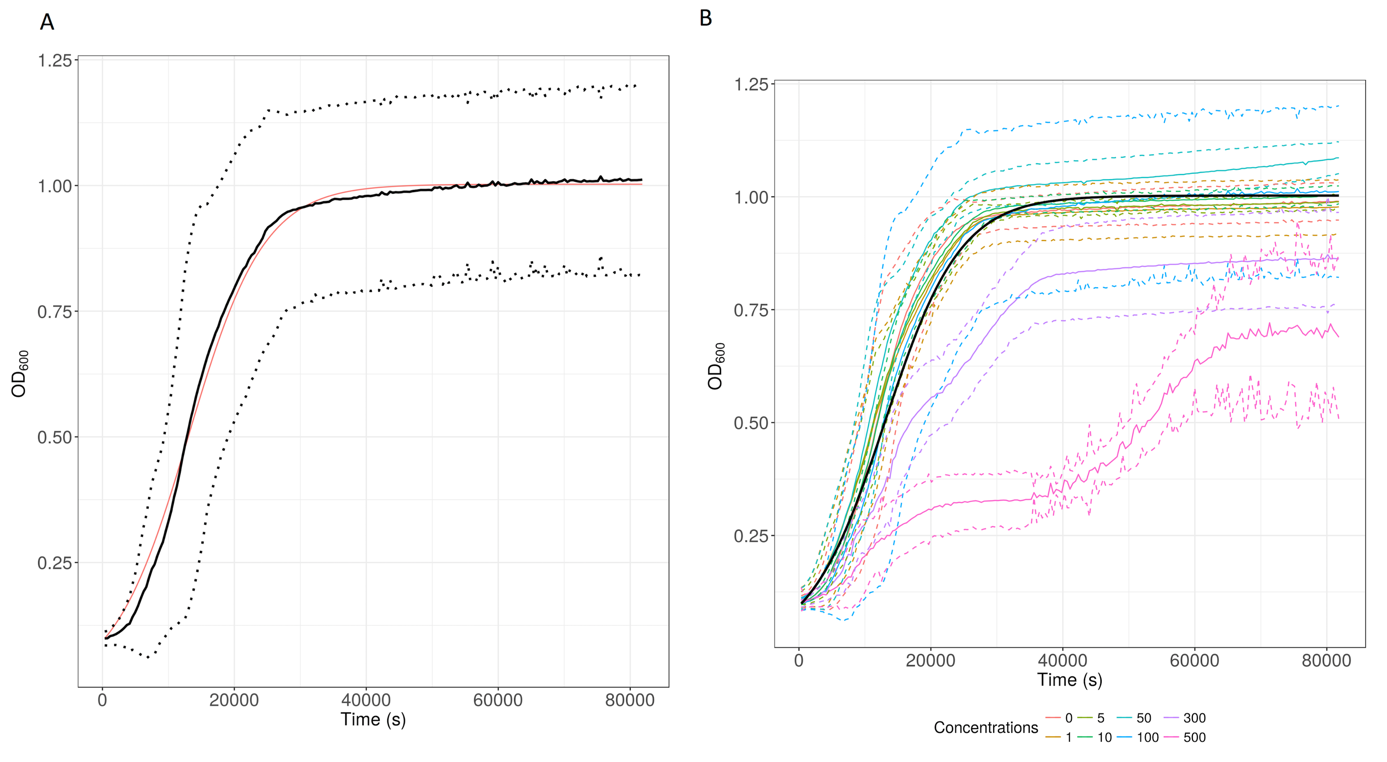
**

**Supplementary figure 4. Growth model fitting to construct 357. (A)** Model (in red) and data (in black) with 95% confidence interval. **(B)** Model (in black) and varying concentrations ranging from 1 µM to 500 µM using the same parameters.

**
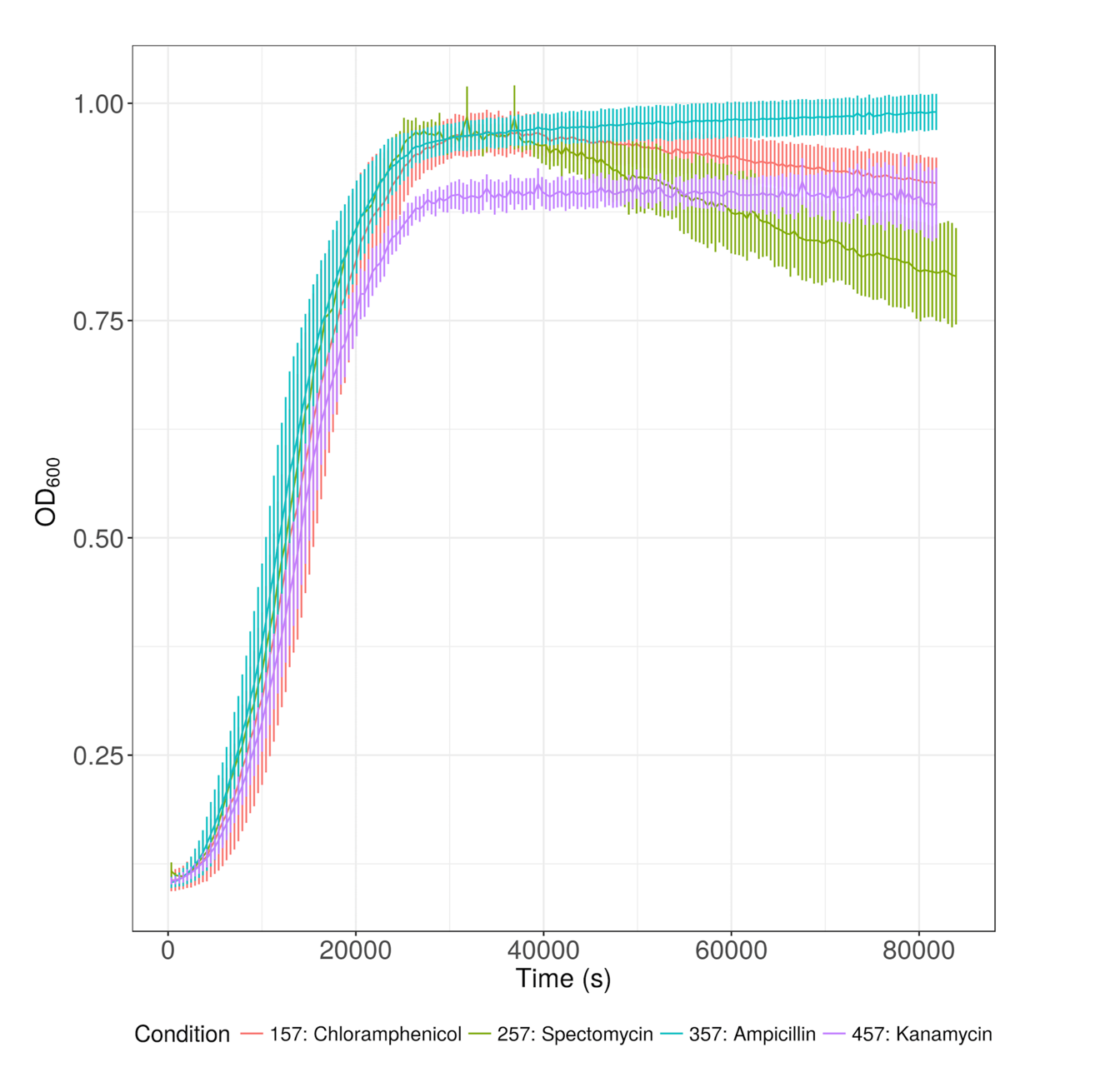
**

**Supplementary figure 5. Growth rate of constructs with varying resistance markers.** Solid lines represent mean of the experimental data and error bars standard deviation. Different colors represent different constructs as indicated on the Figure.

**
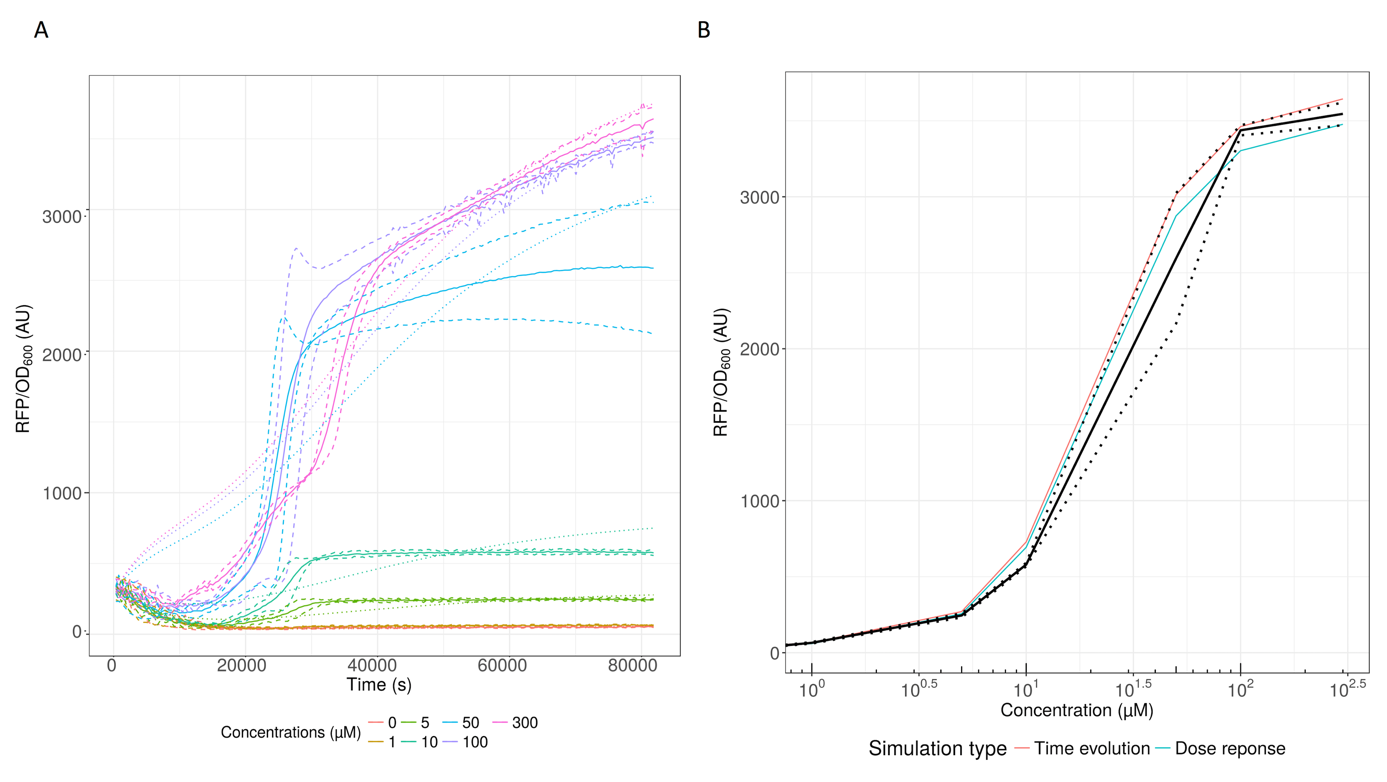
**

**Supplementary figure 6. Time response and dose-response of time-course model for pinocembrin**. **(A)** RFP divided by OD data. Solid lines represent mean of the data and dashed lines 95% confidence intervals. Model is represented as dotted lines. Different colors represent varying concentrations as indicated on the Figure. **(B)** Dose response curve. In black solid line is mean of the data, in black dashed line is the 95% confidence interval. In blue is the model simulating only the dose-response and in red in the model taking into account time evolution.

**
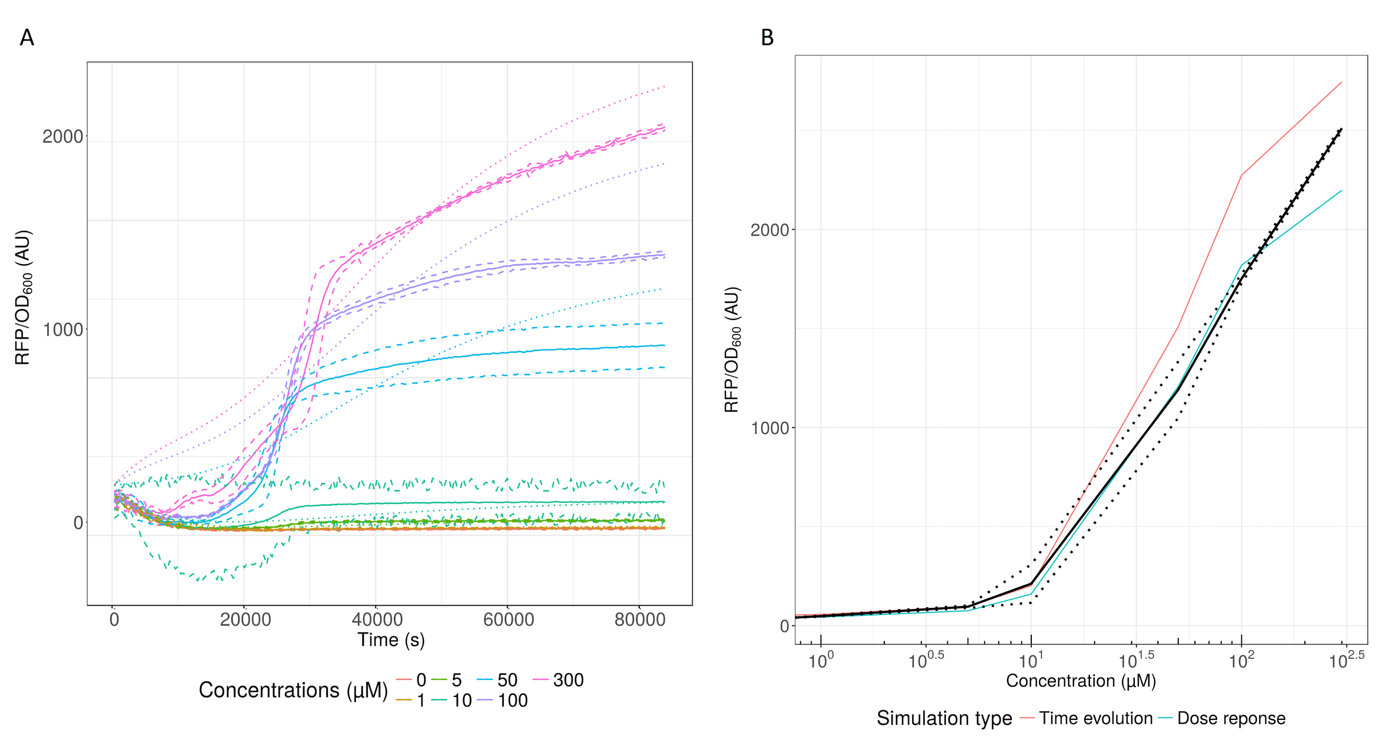
**

**Supplementary figure 7. Time response and dose-response of time-course model for naringenin**. **(A)** RFP divided by OD data. Solid lines represent mean of the data and dashed lines 95% confidence intervals. Model is represented as dotted lines. Different colors represent varying concentrations as indicated on the Figure. **(B)** Dose response curve. In black solid line is mean of the data, in black dashed line is the 95% confidence interval. In blue is the model simulating only the dose-response and in red in the model taking into account time evolution.
