# Supplementary material for: Building a minimal and generalizable model of transcription factor–based biosensors: Showcasing flavonoids
Source: Biotechnol Bioeng. 2018 May 24;115(9):2292–304. doi: 10.1002/bit.26726 (PMC6548992; doi:10.1002/bit.26726)
Supplement: Supplementary file 2 — Supporting information [file BIT-115-2292-s002.docx]

**Building a minimal and generalizable model of transcription-factor based biosensors: showcasing flavonoids**

Heykel Trabelsi,^1^**^*^** Mathilde Koch,^1^**^*^** Jean-Loup Faulon^1,2,3^

Affiliations:

1) Micalis Institute, INRA, AgroParisTech, University Paris-Saclay, 78350 Jouy-en-Josas, FRANCE

2) UMR 8030 Genomics Metabolics, Systems and Synthetic Biology Lab, CEA, CNRS, University of Evry-val-d’Essonne, University Paris-Saclay, Évry, FRANCE

3) SYNBIOCHEM Center, Manchester Institute of Biotechnology, School of Chemistry, University of Manchester, Manchester, UK

*** These authors contributed equally to this work**

Corresponding Author:

E-mail:[jean-loup.faulon@inra.fr](mailto:jean-loup.faulon@inra.fr)

**Supplementary Tables Caption:**

**Supplementary Table I**. Strains and plasmids

**Supplementary Table II**. Primers list

**Supplementary Table III**. Copy numbers of the used plasmids

**Supplementary Table IV**. Pinocembrin-sensor constructs list

**Supplementary Table V**. Parameters, their value and references for the time-course model

**Supplementary Table VI**. Biosensors characteristics

**Supplementary Table I: Strains and plasmids**

|  | **Characteristics** | **Source or Reference** |
| --- | --- | --- |
| **Strains** |  |  |
|  |  |  |
| E. *coli*str. BL21 (DE3) | C> B F^–^ *ompT* *gal* *dcm* *lon* *hsdS_B_*(*r_B_*^–^*m_B_*^–^) λ(DE3 [*lacI* *lacUV5*-*T7p07* *ind1* *sam7* *nin5*]) [*malB*^+^]_K-12_(λ^S^) | Fehér*et al*, (2014). |
| E. *coli* str. DH5α | C>F^–^ *endA1* *glnV44* *thi-1recA1relA1gyrA96deoRnupGpurB20*φ80d*lacZ*ΔM15 Δ(*lacZYA-argF*)U169, hsdR17(*r_K_*^–^*m_K_*^+^), λ^–^ | Fehér*et al*, (2014). |
| Mach1 | ΔrecA1398 endA1 tonA Φ80ΔlacM15 ΔlacX74 hsdR(r_K_^-^ m_K_^+^) | Invitrogen Technology. |
| **Plasmids** |  |  |
|  |  |  |
| pACYC | pACYC Duet / cat + / CmR / P15A rep /LacI | Novagen (EMD Millipore) |
| pCDF | pCDFDUet / aad+ / SpecR / CDF /T7 Prom / LacO / LacI | Novagen (EMD Millipore) |
| pCOLA | pCOLA Duet / kan+ / KanR / ColA / lacI | Novagen (EMD Millipore) |
| pET | pETDuet / bla / ApR / pBR322 / lacI | Novagen (EMD Millipore) |
| pV20 | pSB1A3-FdeR-RFP/ Amp/ FdeR + responsive RFP | This study ( Data not shown) |

**Supplementary Table II: Primers list**

| Primer | 5’->3’ Sequence |
| --- | --- |
| P1 | ACGTCAGGTGGCGCTGACGTCGGTACC |
| P2 | CAACAACGGAGCTCGACCGATGCCCTTGAG |
| P3 | CTCAAGGGCATCGGTCGAGCTCCGTTGTTGTGCTTGTTC |
| P4 | GCTAGCACTGTACCTAGGACTGAGCTAGCCGTCAACTGCAGGAAGACGCAACTAG |
| P5 | CTAGCTCAGTCCTAGGTACAGTGCTAGCCGCCTTTAATATACAAATTTACGTACTCCACTATGCGTTTCAACAAGCTCGAC |
| P6 | CTAGCTCAGTCCTAGGTACAGTGCTAGCTCCCTTTTTAAGCATAGATAAGTAGCCATATTATGCGTTTCAACAAGCTCGAC |
| P7 | CTAGCTCAGTCCTAGGTACAGTGCTAGCACCCTATCTATATATAAGCCTCTAATTCATGCGTTTCAACAAGCTCGAC |
| P8 | CTAGCTCAGTCCTAGGTACAGTGCTAGCACATTTTCACACCTCTCAAGGAGCACACTATGCGTTTCAACAAGCTCGAC |
| P9 | GGTACCGACGTCAGCGCCACCTGACGTCTAAGAAAC |

**Supplementary Table III: Copy numbers of the used plasmids.**

| **Plasmid name** | **Referenced copy number** | **Used copy number** |
| --- | --- | --- |
| **PACYC-Duet** | **10** | **10** |
| **PCDF-Duet** | **20 – 40** | **20** |
| **PET-Duet** | **40** | **40** |
| **PRSF-Duet** | **>100** | **100** |

**Supplementary Table IV: Pinocembrin-sensor constructs list**

| Construct name | Plasmid backbone | Origin of replication | RBS sequence | Resistance Cassette |
| --- | --- | --- | --- | --- |
| 156 | PACYC-Duet | p15A | 1 designed in primer P5) | Chloramphenicol |
| 157 | PACYC-Duet | p15A | 2 designed in primer P6) | Chloramphenicol |
| 158 | PACYC-Duet | p15A | 3 designed in primer P7) | Chloramphenicol |
| 159 | PACYC-Duet | p15A | 4 (designed in primer P8) | Chloramphenicol |
| 256 | PCDF-Duet | CDF | 1 (designed in primer P5) | Spectomycin |
| 257 | PCDF-Duet | CDF | 2 (designed in primer P6) | Spectomycin |
| 258 | PCDF-Duet | CDF | 3 (designed in primer P7) | Spectomycin |
| 259 | PCDF-Duet | CDF | 4 (designed in primer P8) | Spectomycin |
| 356 | PET-Duet | pBR322 | 1 (designed in primer P5) | Ampicillin |
| 357 | PET-Duet | pBR322 | 2 (designed in primer P6) | Ampicillin |
| 358 | PET-Duet | pBR322 | 3 (designed in primer P7) | Ampicillin |
| 359 | PET-Duet | pBR322 | 4 (designed in primer P8) | Ampicillin |
| 456 | PRSF-Duet | RSF | 1 (designed in primer P5) | Kanamycin |
| 457 | PRSF-Duet | RSF | 2 (designed in primer P6) | Kanamycin |
| 458 | PRSF-Duet | RSF | 3 (designed in primer P7) | Kanamycin |
| 459 | PRSF-Duet | RSF | 4 (designed in primer P8) | Kanamycin |

**Supplementary Table V: Parameters, their value and references for the time-course model**.

| **Parameter name** | **Parameter value** | **Parameter description** | **Method of obtention** |
| --- | --- | --- | --- |
| $OD_{0}$ | 0.099 | Starting OD | From data (100 µM of pinocembrin in the construct 357) |
| $OD_{m}$ | 1.00256722590322 ± 0.00211093451610127 | Maximum capacity | Fitted to 100 µM of pinocembrin in the construct 357 |
| k | 0.000174342568009488 ± 1.34152501884726e-06 ($s^{-1}$) | Exponential growth constant of logistic model | Fitted to 100 µM of pinocembrin in the construct 357 |
| k_deg | 3.20464869641221e-05 ($s^{-1}$) | Degradation constant | Fitted to 100 µM of pinocembrin in the construct |
| alpha | 0.00193734987003592 (AU) | Term accounting for production and fluorescence | Fitted to 100 µM of pinocembrin in the construct 357 |

**Supplementary Table VI: Biosensors characteristics.**

| **Compound** | **Plasmid construct** | **n** | **ratio** | **IC50 (or K_d_)** |
| --- | --- | --- | --- | --- |
| pinocembrin | 156 | 1.63± 0.12 | 56.62± 2.24 | 71.02± 5.57 |
| naringenin | 156 | 1.49 ± 0.04 | 42.52 ± 0.64 | 49.30 ± 2.18 |
| pinocembrin | 157 | 1.89 ± 1.26 | 1.27 ± 0.24 | 65.87 ± 24.16 |
| naringenin | 157 | 1.77 ± 0.34 | 1.14 ± 0.05 | 43.47 ± 5.93 |
| pinocembrin | 159 | 2.04 ± 0.18 | 46.95 ± 0.82 | 27.97 ± 2.45 |
| naringenin | 159 | 1.51 ± 0.06 | 37.66 ± 0.90 | 50.63 ± 2.71 |
| pinocembrin | 257 | 1.56 ± 0.12 | 17.70 ± 0.80 | 73.68 ± 6.41 |
| naringenin | 257 | 1.68 ± 0.45 | 6.02 ± 0.37 | 53. 25± 5.86 |
| pinocembrin | 357 | 1.96 ± 0.20 | 63.24 ± 1.48 | 23.07 ± 2.38 |
| naringenin | 357 | 1.51 ± 0.07 | 61.57 ± 1.09 | 58.82 ± 2.51 |
| pinocembrin | 457 | 1.92 ± 0.17 | 42.37 ± 1.38 | 9.62 ± 0.66 |
| naringenin | 457 | 1.91 ± 0.57 | 30.17 ± 4.59 | 22.53 ± 7.62 |

**
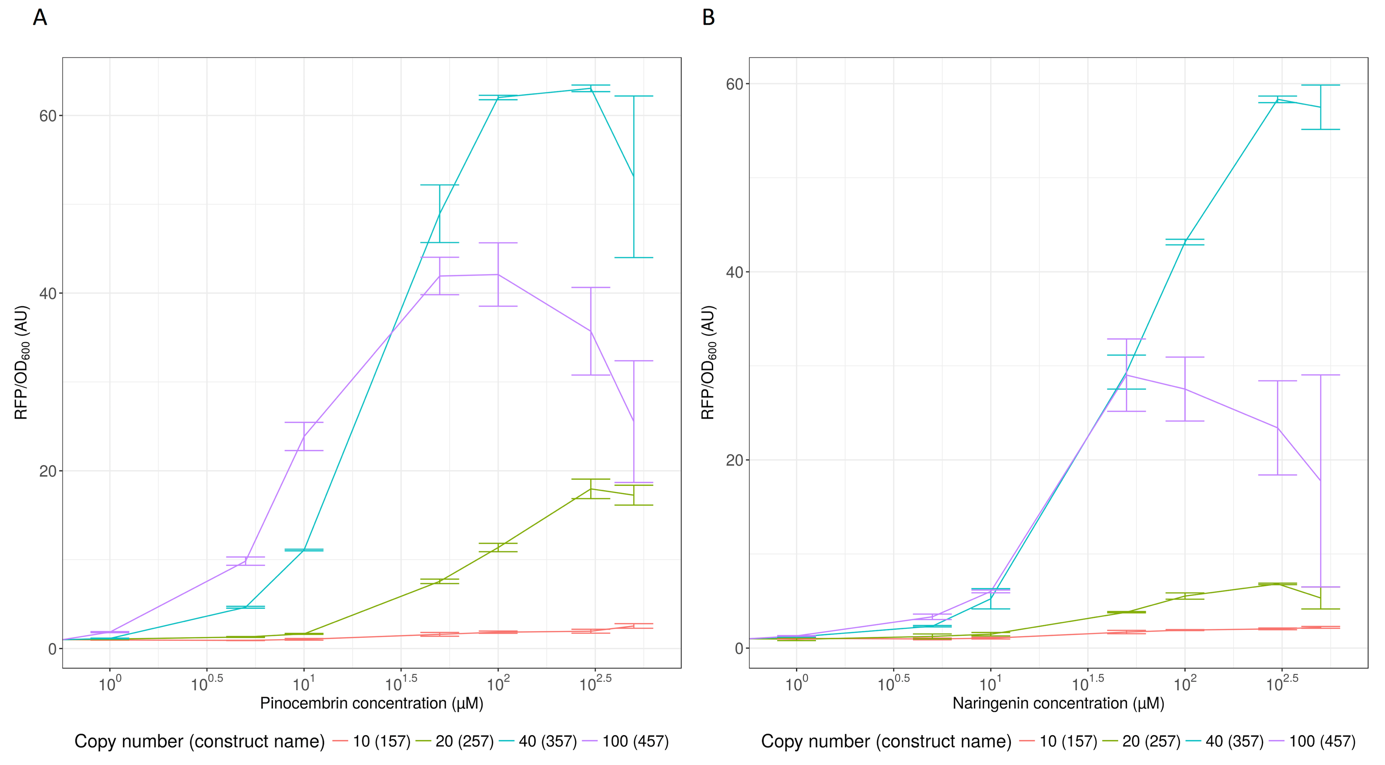
**

**Supplementary figure 1. Effect of copy number variation on data fold change for pinocembrin (A) and naringenin (B).** In colors are represented the different constructs. The assumed copy numbers are as follows: 10 for 157, 20 for 257, 40 for 357 and 100 for 457. Concentration of the inducer are expressed in µM and vary from 0 to 500. Error bars represent standard deviation.

**
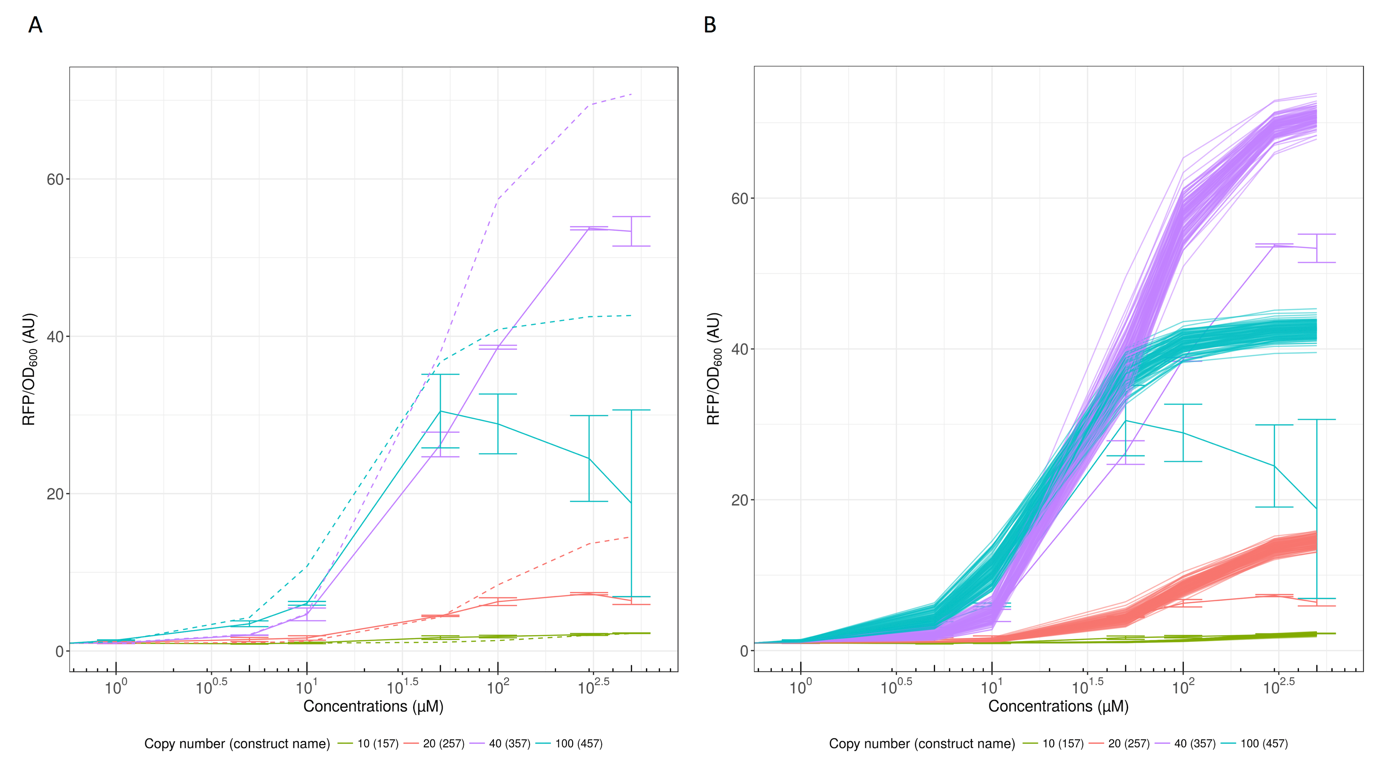
**

**Supplementary figure 2. Model fitting to naringenin data for varying copy number without correcting parameter. (A)** Best fit parameters for naringenin. **(B)** 100 random simulations from parameters fitting for naringenin. Error bars represent standard deviation.
